# Development of Novel Pharmaceutical Forms of the Marine Bioactive Pigment Echinochrome A Enabling Alternative Routes of Administration

**DOI:** 10.3390/md21040250

**Published:** 2023-04-18

**Authors:** Stefanos Kikionis, Paraskevi Papakyriakopoulou, Panagiotis Mavrogiorgis, Elena A. Vasileva, Natalia P. Mishchenko, Sergey A. Fedoreyev, Georgia Valsami, Efstathia Ioannou, Vassilios Roussis

**Affiliations:** 1 Section of Pharmacognosy and Chemistry of Natural Products, Department of Pharmacy, National and Kapodistrian University of Athens, Panepistimiopolis Zografou, 15771 Athens, Greece; skikionis@pharm.uoa.gr (S.K.); panagiotismavro1991@gmail.com (P.M.); eioannou@pharm.uoa.gr (E.I.); 2 Section of Pharmaceutical Technology, Department of Pharmacy, National and Kapodistrian University of Athens, Panepistimiopolis Zografou, 15784 Athens, Greece; ppapakyr@pharm.uoa.gr; 3 Laboratory of the Chemistry of Natural Quinonoid Compounds of the G. B. Elyakov Pacific Institute of Bioorganic Chemistry, Far-Eastern Branch of the Russian Academy of Science, Prospect 100 let Vladivostoku, 159, 690022 Vladivostok, Russia; vasilieva_el_an@mail.ru (E.A.V.); mischenkonp@mail.ru (N.P.M.); fedoreev-s@mail.ru (S.A.F.)

**Keywords:** Echinochrome A, micro-/nanofibers, electrospinning, controlled release, dissolution, permeability

## Abstract

Echinochrome A (EchA), a marine bioactive pigment isolated from various sea urchin species, is the active agent of the clinically approved drug Histochrome^®^. EchA is currently only available in the form of an isotonic solution of its di- and tri-sodium salts due to its poor water solubility and sensitivity to oxidation. Electrospun polymeric nanofibers have lately emerged as promising drug carriers capable of improving the dissolution and bioavailability of drugs with limited water solubility. In the current study, EchA isolated from sea urchins of the genus *Diadema* collected at the island of Kastellorizo was incorporated in electrospun micro-/nanofibrous matrices composed of polycaprolactone and polyvinylpyrrolidone in various combinations. The physicochemical properties of the micro-/nanofibers were characterized using SEM, FT-IR, TGA and DSC analyses. The fabricated matrices exhibited variable dissolution/release profiles of EchA, as evidenced in in vitro experiments using gastrointestinal-like fluids (pH 1.2, 4.5 and 6.8). Ex vivo permeability studies using the EchA-loaded micro-/nanofibrous matrices showed an increased permeation of EchA across the duodenum barrier. The results of our study clearly show that electrospun polymeric micro-/nanofibers represent promising carriers for the development of new pharmaceutical formulations with controlled release, as well as increased stability and solubility of EchA, suitable for oral administration, while offering the potential for targeted delivery.

## 1. Introduction

Echinochrome A (7-ethyl-2,3,5,6,8-pentahydroxy-1,4-naphthoquinone, EchA) is a marine pentahydroxyethyl naphthoquinone and is the most abundant pigment found in various sea urchin species [1]. It is isolated as a red crystalline powder (Russian Federation registration number P N002362/01-2003), and, in the form of its sodium salts, it is used as the active ingredient of the commercially available drug Histochrome^®^ [2]. Histochrome^®^ (Russian Federation registration number P N002363/01-2003) is used as a cardioprotective and antioxidant drug for the treatment of various cardiovascular diseases, such as coronary heart disease and reduction of the necrotic zone in myocardial infarction [3]. Histochrome^®^ (Russian Federation registration number P N002363/02-2003) is also used in ophthalmology for the treatment of ocular diseases, such as macular degeneration, cornea and retina degenerative diseases, primary open-angle glaucoma, post-traumatic hemorrhages, diabetic retinopathy and dyscirculatory disorders in the central artery and vein of the retina [4,5].

EchA can simultaneously block a number of free radical reactions by the neutralization of reactive oxygen species, nitric oxide and peroxide radicals, the chelation of metal ions, the inhibition of lipid peroxidation and the regulation of antioxidant enzymes’ levels [6,7]. Over the last years, there has been an increasing interest in the expansion of the commercial applications of EchA since, besides its antioxidant and cardioprotective activities [8,9,10,11,12], EchA has been reported to exhibit anti-inflammatory [13,14,15,16], antiviral [17] and antibacterial [18] activities, among others [19,20,21,22,23,24,25]. Recently, the multifaceted clinical effects and the mechanisms of action of EchA in the treatment of various cardiovascular, ophthalmic, cerebrovascular, metabolic and inflammatory diseases have been described, highlighting its remarkable pharmacological activities and therapeutic potential [26].

Currently, EchA is used only in the form of an isotonic solution of its di- and tri-sodium salts. The poor water solubility of EchA, in combination with its low stability in solution due to its sensitivity to oxidation, restrict its use in the pharmaceutical sector, prohibiting, up to now, its oral, buccal, nasal or transdermal administration.

Water solubility is a crucial parameter in drug formulation, influencing drug pharmacodynamics and pharmacokinetics. Most of the failures in the development of new drugs are usually associated with the poor water solubility of the active ingredient and the low stability of its solutions. Poor solubility can lead to low bioavailability and, as a result, to suboptimal drug delivery and low efficacy. About 40% of the drugs in the market and almost 90% of drug candidate molecules are poorly soluble in water [27]. Taking into account the range of activities displayed by EchA, the development of new formulations that could increase its solubility and enhance its stability while maintaining its efficacy is of significance. Recently, complexes of β-cyclodextrin–histochrome [28] and carrageenan–EchA [29,30,31] exhibiting increased stability/solubility in aqueous media have been reported as promising pharmaceutical forms of EchA.

Polymeric micro-/nanofibers have attracted significant interest in drug delivery applications as suitable drug carriers of both water-soluble and poorly water-soluble drugs due to their high encapsulation efficiency and high loading capacity [32,33,34]. Fibrous scaffolds can be easily produced from electrically charged polymeric solutions or melts by electrospinning, the most efficient, scalable and versatile technique for the preparation of polymeric fibers with diameters ranging from the submicron down to the nanometer scale [35,36,37]. Various active pharmaceutical ingredients can be incorporated into electrospun micro-/nanofibers for the preparation of alternative solid forms with modified and controlled release characteristics [38,39,40]. The rapid evaporation of solvents during electrospinning facilitates the incorporation of active ingredients into fibrous networks in the amorphous physical state. Due to homogeneous drug distribution and restricted molecular motion within the polymer matrix, electrospun micro-/nanofibers can maintain the embedded poorly water-soluble drugs in an amorphous state for prolonged periods, improving drug dissolution and bioavailability [41,42].

In the framework of our research interests towards the development of electrospun micro-/nanofibrous matrices for drug delivery and other biomedical applications [43,44,45,46], we invested in the development of new pharmaceutical formulations of EchA through its incorporation in polymeric micro-/nanofibers. Electrospun micro-/nanofibrous matrices composed of polycaprolactone (PCL) or/and polyvinylpyrrolidone (PVP) in various ratios loaded with EchA were fabricated and characterized by SEM, FT-IR, TGA and DSC analyses. The dissolution/release profile of EchA from the electrospun matrices was evaluated in vitro in gastrointestinal-like fluids, while the permeation of EchA across the duodenum barrier was assessed in ex vivo permeability studies on the small intestine of young rabbits.

## 2. Results and Discussion

Micro-/nanofibers represent alternative drug delivery systems that allow for the encapsulation and controlled release of various active ingredients. In the present study, EchA was isolated from sea urchins of the genus *Diadema* collected at the island of Kastellorizo and successfully incorporated into electrospun micro-/nanofibers composed of PCL or/and PVP at different ratios that were evaluated for their efficacy to serve as controlled release systems of EchA suitable for oral administration.

### 2.1. Physicochemical Characterization of Electrospun Micro-/Nanofibrous Patches

PCL and PVP were selected to serve as EchA-polymer carriers since they are non-toxic polymers that can be easily electrospun, offering, due to their high hydrophobic and hydrophilic character, respectively, different release profiles. In total, six different micro-/nanofibrous matrices incorporating in all cases EchA in a 10% *w/w* (weight to matrix weight) final concentration were fabricated, including two scaffolds based solely on PCL or PVP (PCL-EchA and PVP-EchA, respectively), three blended fiber mats resulting from the co-electrospinning of PCL and PVP in different ratios in an antiparallel setup [PCL-EchA/PVP-EchA (1:3), PCL-EchA/PVP-EchA (1:1) and PCL-EchA/PVP-EchA (3:1)] and one composite fiber mat resulting from the electrospinning of a single spinning solution of PCL and PVP [[PCL-PVP(1:3)]-EchA].

The chemical integrity of EchA following electrospinning was verified by ^1^H NMR and UV-Vis analyses of the recovered compound after extraction of the fabricated fiber mats.

**Figure 1:**
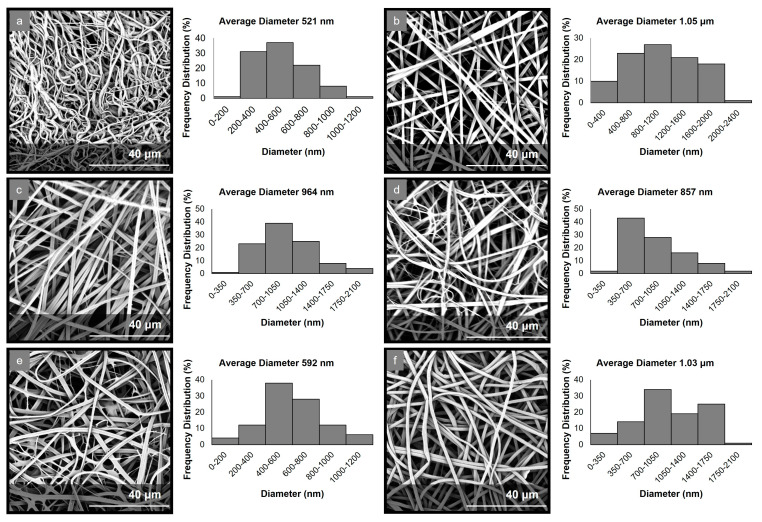
SEM images and diameter distribution histograms of (**a**) PCL-EchA, (**b**) PVP-EchA, (**c**) PCL-EchA/PVP-EchA (1:3), (**d**) PCL-EchA/PVP-EchA (1:1), (**e**) PCL-EchA/PVP-EchA (3:1) and (**f**) [PCL-PVP(1:3)]-EchA fibers.

The electrospinning parameters were optimized to allow for the production of uniform fibrous matrices with bead-free fibers in all cases. The morphological characteristics of the fabricated micro-/nanofibrous matrices were evaluated through analyses of the obtained SEM images (Figure 1). The examination of the PCL-EchA fiber mat revealed a network of cylindrical-shaped fibers with diameters ranging from 185 nm to 1.15 μm and an average diameter size of 521 ± 96 nm. In the case of the PVP-EchA fiber mat, smooth fibers of a cylindrical morphology and a higher diameter size were observed, with diameters ranging from 24 nm to 2.28 μm and an average diameter size of 1.05 ± 0.16 μm. The PCL-EchA/PVP-EchA (1:3), PCL-EchA/PVP-EchA (1:1) and PCL-EchA/PVP-EchA (3:1) blended fiber mats exhibited similar homogeneous fibrous networks with the fiber size differences attributed to the different feeding rates of the PCL-EchA and PVP-EchA spinning solutions during the electrospinning process and the different ratio of the corresponding blended fibers. The PCL-EchA/PVP-EchA (1:3) fiber mat consisted of fibers with diameters ranging from 77 nm to 1.92 μm with an average diameter size of 964 ± 183 nm, whereas in the case of the PCL-EchA/PVP-EchA (1:1) fiber mat, the fiber diameters measured from 105 nm to 1.92 μm with an average diameter size of 857 ± 150 nm. In the case of PCL-EchA/PVP-EchA (3:1), the mat fibers’ diameters ranged from 34 nm to 1.16 μm with an average diameter size of 592 ± 115 nm. A uniform micro-/nanofibrous network with the diameters of the fibers ranging from 78 nm to 1.87 μm and an average diameter size of 1.03 ± 0.16 μm was also observed in the case of the composite [PCL-PVP(1:3)]-EchA fiber mat, indicating that the combination of the polymers in the same spinning solution did not affect their spinability.

**Figure 2:**
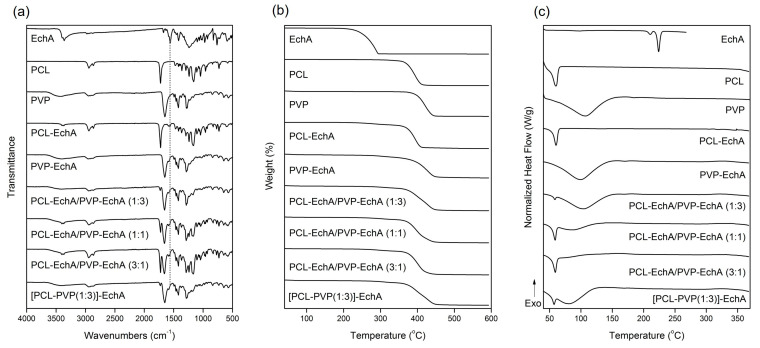
(**a**) FT-IR spectra, (**b**) TGA and (**c**) DSC thermograms of EchA, PCL, PVP, PCL-EchA, PVP-EchA, PCL-EchA/PVP-EchA (1:3), PCL-EchA/PVP-EchA (1:1), PCL-EchA/PVP-EchA (3:1) and [PCL-PVP(1:3)]-EchA fibrous matrices.

The FT-IR spectrum of EchA included a broad absorption band centered approximately at 3363 cm^−1^ assigned to –OH stretching vibrations, a –C=C stretching at 1675 cm^−1^, a characteristic carbonyl –C=O absorption band at 1560 cm^−1^ and in-plane bending vibrations of the hydroxyl groups at 1420 cm^−1^ (Figure 2a) [29]. In the FT-IR spectrum of PCL, the absorption bands observed at 2944 and 2867 cm^−1^ were attributed to asymmetric and symmetric –CH_2_ stretching vibrations, whereas the carbonyl –C=O stretching was recorded at 1725 cm^−1^ [47]. The FT-IR spectrum of PVP exhibited a characteristic broad absorption band at 3434 cm^−1^ assigned to –OH stretching vibrations and bands at 2948 and 1651 cm^−1^ attributed to –CH_2_ and –C=O stretching vibrations, respectively. –C–H and –C–N bending vibrations were recorded at 1422 and 1284 cm^−1^, respectively, whereas the –N–C=O bending was observed at 570 cm^−1^ [48].

The FT-IR spectra of the fabricated matrices revealed the characteristic signals of their ingredients (Figure 2a). Due to the large amount of PCL and PVP dominating the fibrous matrices, all scaffolds exhibited mainly the characteristic absorption bands of the polymeric components. The incorporation of EchA into the polymeric fibers was evident from the absorption band at 1560 cm^−1^ attributed to carbonyl –C=O stretching vibrations since the other signals overlapped with those of PCL and/or PVP. The PCL-EchA/PVP-EchA (1:3), PCL-EchA/PVP-EchA (1:1), PCL-EchA/PVP-EchA (3:1) and [PCL-PVP(1:3)]-EchA fiber mats exhibited similar absorption bands in their FT-IR spectra, with the intensity of the –C=O band of PCL at 1725 cm^−1^ being analogous to the proportion of PCL in the fiber mats.

The physicochemical properties of the fabricated micro-/nanofibrous patches, as well as those of the utilized raw materials, were characterized by TGA and DSC analyses. As shown in the TGA thermograms (Figure 2b), EchA started to decompose at 220 °C, and its degradation was completed at approx. 294 °C. The thermal decomposition of PCL was observed between 355 and 411 °C, whereas PVP was recorded to decompose between 373 and 445 °C. The thermogravimetric curves of the designed matrices revealed the synergistic degradation phenomena of the combined ingredients. The PCL-EchA fibers started to decompose at 248 °C due to the degradation of EchA, with their main mass loss occurring from 357 °C until complete degradation at around 411 °C. In the case of PVP-EchA, the fibers started to decompose at 282 °C, whereas the main decomposition of the fiber mat occurred from 371 to 449 °C. The different ratios of the combined polymers in the blended and composite fibers resulted in different thermal degradation patterns for the corresponding matrices. In the case of the blended fiber mats, the PCL-EchA/PVP-EchA (1:3) scaffold started to decompose at 289 °C due to the presence of EchA, and its main degradation occurred between 359 and 448 °C. In the case of the PCL-EchA/PVP-EchA (1:1) fiber mat, the presence of EchA initiated the decomposition of the fibers at 275 °C, with the main mass loss occurring from 360 to 444 °C. The PCL-EchA/PVP-EchA (3:1) fiber mat started to decompose at 261 °C due to the degradation of EchA, and its major decomposition was recorded between 360 and 441 °C. The decomposition of the composite [PCL-PVP(1:3)]-EchA fiber mat was initiated at 285 °C due to the decomposition of EchA, and its main degradation occurred from 361 to 448 °C.

The DSC thermogram of EchA revealed a weak endotherm at 210.5 °C and a sharp endothermic peak at 223.9 °C attributed to the melting of the compound (Figure 2c). PCL exhibited a sharp melting endotherm at 59.9 °C and PVP showed a broad dehydration endotherm at 105.9 °C. In the thermograms of the various micro-/nanofibrous matrices, only the sharp melting peak of PCL and the broad dehydration curve of PVP were clearly evident at slightly shifted temperatures and different curve patterns due to different synergistic thermal events were attributed to their different compositions, fabrication methods and structural fiber characteristics. None of the characteristic thermal events of EchA were evident in the thermograms of the micro-/nanofiber matrices, indicating the absence of crystalline EchA within the fabricated matrices due to its conversion to an amorphous state during electrospinning [49].

### 2.2. Dissolution Studies

In vitro dissolution testing is widely used to assess the drug release of developed pharmaceutical products. According to the European Medicine Agency (EMA) [50] and the US Food and Drug Administration (FDA) [51] guidelines for solid oral dosage forms, dissolution testing should be performed in aqueous media with a pH range from 1.2 to 6.8. More specifically, the effect of the dissolution medium on the in vitro drug release should be evaluated at three different pH values (1.2, 4.5 and 6.8), corresponding to the different pH conditions that the drug undergoes while traversing the gastrointestinal tract. Therefore, all developed micro-/nanofibrous patches, as well as neat EchA, were tested at pH 1.2, 4.5 and 6.8 using a USP dissolution apparatus-II with the paddle method.

**Figure 3:**
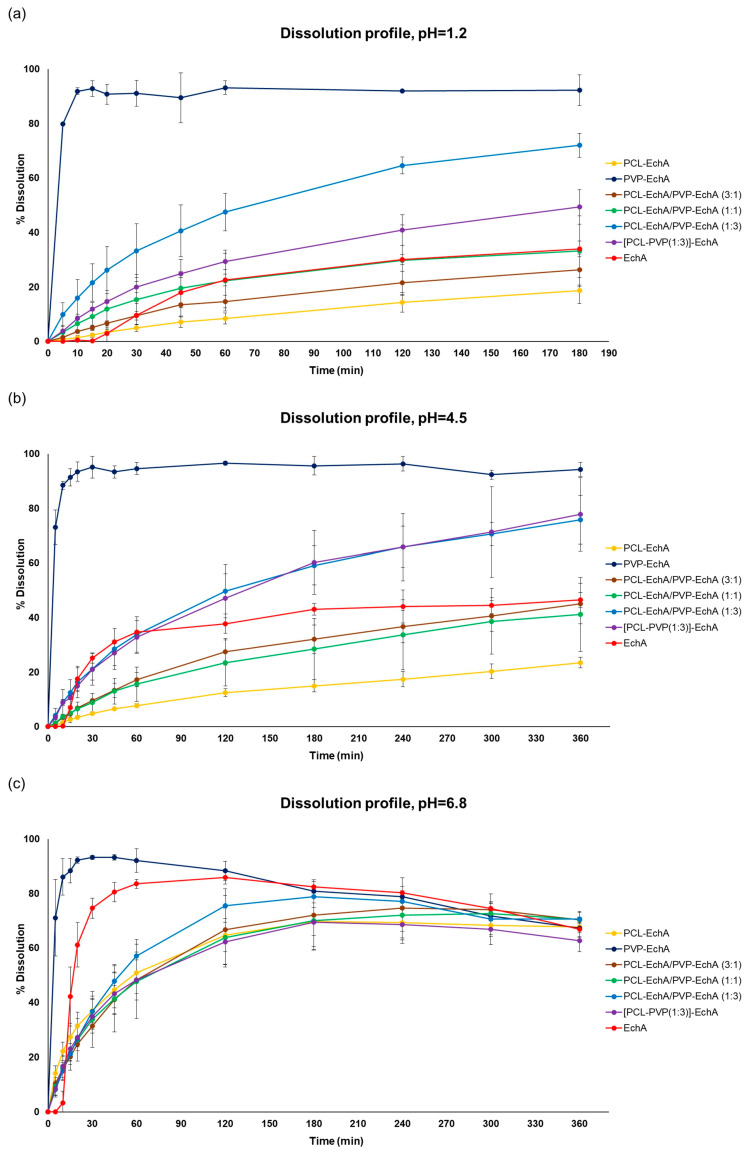
Dissolution/release profiles of neat EchA and PCL-EchA, PVP-EchA, PCL-EchA/PVP-EchA (3:1), PCL-EchA/PVP-EchA (1:1), PCL-EchA/PVP-EchA (1:3) and [PCL-PVP(1:3)]-EchA fibrous matrices at pH (**a**) 1.2, (**b**) 4.5 and (**c**) 6.8 (mean ± SD, *n* = 3).

In all tested dissolution media, the PVP-EchA fiber mats showed a burst release of EchA corresponding to 91.1 ± 4.7, 95.1 ± 4.0 and 93.3 ± 0.7% of the loading dose at pH 1.2, 4.5 and 6.8, respectively (Figure 3). These release values are reported for the 30 min time point, indicating a pH-independent release of EchA that rapidly occurred in all three dissolution media. The conversion of EchA to an amorphous state during the electrospinning process, as suggested by the DSC thermograms, enabled a higher solubility of EchA released from the fibrous scaffolds in comparison to that of neat EchA at all tested pH conditions, as demonstrated by the performance of the PVP-EchA fiber mats. Specifically, after the immediate release of EchA during the deconstruction of the PVP fiber mats at the time point of 30 min, no precipitation of the substance was observed in any of the pH conditions. The high hydrophilicity of PVP enabled the effective wetting of the fiber mat by the water molecules [52] and allowed the immediate release of the incorporated EchA, which remained dissolved in the dissolution media. On the contrary, the lipophilic PCL employed in the case of the PCL-EchA fiber mats impeded the hydration of the scaffold, retarding the release of EchA, as depicted in the dissolution profiles at pH 1.2 and 4.5 (Figure 3).

At pH 1.2 and 4.5, all patches containing PCL showed sustained release profiles. The percentage of EchA released from the blended PCL-EchA/PVP-EchA fiber mats at both pH values increased gradually as the ratio of PVP/PCL in the patch increased. At pH 1.2, this trend was obvious when comparing PCL-EchA to PCL-EchA/PVP-EchA (3:1), PCL-EchA/PVP-EchA (1:1) and PCL-EchA/PVP-EchA (1:3), even though the level of variance in some cases resulted in a non-significant statistical difference (Figure 3a). It is worth noting that at pH 1.2, the blended PCL-EchA/PVP-EchA (1:3) performed markedly better than the composite [PCL-PVP(1:3)]-EchA mats, displaying almost double the release rate of the latter, even though the two fibrous scaffolds differed only in the method of fabrication. On the contrary, at pH 4.5, both fibrous scaffolds displayed similar dissolution profiles (Figure 3b).

EchA is known to be more soluble in higher pH values, as was also observed in the current study (Figure 3c). In particular, the dissolution of neat EchA at pH 6.8 approached the fast-release profile of the PVP-EchA mats, while the PCL-containing patches maintained the sustained release that was also observed at pH 1.2 and 4.5. The lower stability of EchA at less-acidic pH conditions led to a gradual degradation of EchA from the 60 min time point of the dissolution experiment and onwards. When PCL was present in the nanofibrous formulations at an equal or greater ratio to PVP (PCL-EchA, PCL-EchA/PVP-EchA (3:1) and PCL-EchA/PVP-EchA (1:1) fiber mats), the degradation of EchA was slightly delayed due to its slower release rate from the hydrophobic PCL-dominated scaffolds. At pH 6.8, the dissolution profiles of the PCL-containing mats were not significantly different at all time points of the study. The similar trend observed for their dissolution profiles at pH 6.8 may point to an effect of the pH on the polymer’s integrity, as also reported in the literature [53]. Indeed, the degradation process of PCL has been found to be more favored as the medium environment becomes more alkaline due to OH^-^ nucleophilic substitution on the carbonyl group [54,55]. Accordingly, the faster release of EchA from the PCL-containing mats at pH 6.8 can probably be attributed to a possible alteration of the polymer’s structure at this pH value.

### 2.3. Permeability Studies

The dissolution studies revealed that the combination of two polymers, one with a high hydrophilicity and one highly lipophilic, led to the development of fibrous scaffolds that offered a controlled release of EchA. The release rate was determined by the ratio of the two polymers, being higher when the amount of PVP prevailed. The burst release of EchA from the PVP-EchA mats was not considered desirable since the active compound is mostly absorbed in the intestine. Therefore, the fast release in the acidic conditions of the stomach would probably favor the degradation of EchA, without significantly contributing to the absorption process. Accordingly, the PCL-EchA/PVP-EchA (1:3) and the [PCL-PVP(1:3)]-EchA fiber mats, fabricated as either blended or composite, respectively, were selected to be tested in an ex vivo permeability study using rabbit intestine tissues as the biological barrier.

**Figure 4:**
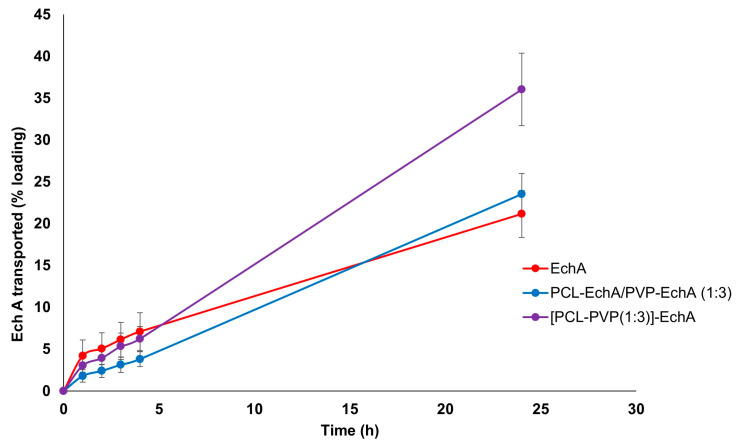
Permeation profiles of EchA from the PCL-EchA/PVP-EchA (1:3) and [PCL-PVP(1:3)]-EchA fibrous matrices, as well as that of neat EchA, through the intestine barrier of rabbits, expressed as quantity permeated per unit area (mean ± SD, *n* = 7).

The ex vivo permeation experiments with PCL-EchA/PVP-EchA (1:3) and [PCL-PVP(1:3)]-EchA patches were performed in the duodenum, the most proximal portion of the small intestine. The permeability of neat EchA was also assessed as a control in order to be compared with the values obtained from the fibrous mats. During the first four hours of the study, no significant differences were noted among the tested formulations. However, at the final time point of the 24 h, the amount of EchA transported across the intestine barrier was higher in the case of the composite [PCL-PVP(1:3)]-EchA patch compared to that of the blended PCL-EchA/PVP-EchA (1:3) mat and neat EchA. Specifically, the permeation from the [PCL-PVP(1:3)]-EchA patch, expressed as a % amount of the loading dose of EchA, was found to be 50% higher than that of both the PCL-EchA/PVP-EchA (1:3) mat and neat EchA (Figure 4).

The mixing of polymers in a single spinning solution electrospun towards the fabrication of the composite [PCL-PVP(1:3)]-EchA fibrous mats led to surfaces of uniform fibers composed of both hydrophilic and lipophilic parts. In contrast, the antiparallel electrospinning employed for the fabrication of the blended PCL-EchA/PVP-EchA (1:3) scaffold was expected to result in a network of PCL and PVP fibers. This structural difference between the two formulations could also be observed in the SEM images (Figure 1), which depicted different architectures for the two nanofibrous mats that may explain their different ex vivo performances. As previously reported [56], PCL presents adequate mucoadhesion at the beginning of the intestine mediated by hydrophobic interactions. Thus, the presence of PCL in all fibers of the [PCL-PVP(1:3)]-EchA mat would probably allow a more efficient interaction of the composite fibers with the intestinal mucosa, as was also revealed by the higher % of EchA transported across the intestine barrier. On the contrary, the hydrophilicity of the PVP fibers that dominated the structure of the PCL-EchA/PVP-EchA (1:3) patch and favored its dissolution, as established by the in vitro experiments, may render the meshing of PCL with the mucus layer more difficult. It is important to note that the structure of the mucosa affects the diffusivity and partition of EchA, evidencing the differences between the formulations that could not be observed in the in vitro dissolution experiments.

In terms of EchA stability, the higher mass balance of the PCL-EchA/PVP-EchA (1:3) and the [PCL-PVP(1:3)]-EchA patches indicated a protective effect of PCL against the pH-induced degradation. Specifically, the mass balance, calculated as the sum of EchA in the receptor, donor and mucosa, was equal to 42.57 ± 8.44% in the case of neat EchA, which was markedly increased when EchA was formulated in the fiber mats, as revealed by the 62.54 ± 9.43 and 70.11 ± 17.6% recovery in the cases of the PCL-EchA/PVP-EchA (1:3) and [PCL-PVP(1:3)]-EchA patches, respectively.

According to our results, it is evident that the selection of the polymers used as carriers of EchA can affect the release profile, allowing for the preparation of micro-/nanofibrous patches according to the desired properties for different routes of administration, while holding the potential for targeted delivery.

## 3. Materials and Methods

### 3.1. Materials

Polycaprolactone (PCL) (Mw 80,000), polyvinylpyrrolidone (PVP) (Mw 1,300,000), dichloromethane (DCM), dimethylformamide (DMF) and ethanol (EtOH) were purchased from Sigma-Aldrich (Darmstadt, Germany). All chemicals were of reagent grade and used directly without further purification. Disodium hydrogen phosphate and monosodium phosphate were acquired from Merck KGaA (Darmstadt, Germany). Sodium citrate dihydrate and citric acid were purchased from Fischer (Waltham, MA, USA). Triple-deionized water was used for all preparations. Clear hydroxypropyl methylcellulose, capsules size 0, were purchased from Capsule Fillers (Bologna, Italy). Normal-phase and reversed-phase C_18_ silica gel for column chromatography and TLC plates with Kieselgel 60 F_254_ and RP-18 F_254s_ on aluminum support were purchased from Merck (Darmstadt, Germany).

### 3.2. Biological Material

Sea urchins of the genus *Diadema* were collected by SCUBA diving at a depth of 5–7 m, at Agios Georgios bay, Kastellorizo Island, Greece, in September 2021. The urchins were immediately frozen, transferred to the Section of Pharmacognosy and Chemistry of Natural Products, Department of Pharmacy, National and Kapodistrian University of Athens, and stored at –20 °C until analyzed.

### 3.3. Extraction and Isolation of Echinochrome A

After washing with cold fresh water, the sea urchins were dissected, and the entrails were removed. The shells and spines (7.5 kg) were crushed into small pieces and exhaustively extracted with an ethanol solution (70% *v/v*) containing sulfuric acid (8–10% *v/v*) at room temperature for 24 h three times. Subsequently, the acidified extract was filtered and concentrated under reduced pressure to afford a viscous residue which was diluted with distilled water and sequentially extracted with CH_2_Cl_2_ and EtOAc. After evaporation of the organic solvents in vacuo, the CH_2_Cl_2_ and EtOAc extracts were pooled together and subjected to vacuum column chromatography over silica gel acidified with oxalic acid in ethanol, using mixtures of cHex with increasing amounts of CH_2_Cl_2_ and subsequently CH_2_Cl_2_ with increasing amounts of MeOH as the mobile phase, to yield 8 fractions (A–H). Fraction F (12 g) was subjected to vacuum column chromatography over C_18_ silica gel, using H_2_O with increasing amounts of MeOH and finally EtOAc as eluent, to yield 12 fractions (F1–F12). Fraction F8 (H_2_O/MeOH 20:80, 4 g) was further purified using vacuum column chromatography over C_18_ silica gel, using H_2_O with increasing amounts of MeOH as the mobile phase, to yield 8 fractions (F8a–F8h). Fraction F8d (H_2_O/MeOH 20:80, 2.95 g) was identified as EchA in pure form by comparison of its spectroscopic and physical data with those reported in the literature [57].

### 3.4. Preparation of Electrospun Micro-/Nanofibrous Patches

Electrospinning was conducted using a *γ*-High Voltage Research DC power supply generator (Gamma High Voltage Research, Ormond Beach, FL, USA) with the spinning solutions being loaded into 10 mL disposable syringes fitted with stainless steel blunt needles (23G). The syringes were mounted on a horizontally positioned programmable syringe pump (Harvard PHD 2000, Harvard Apparatus, Holliston, MA, USA), and the produced micro-/nanofibers were deposited on aluminum foil wrapped on an RC-6000 (NaBond Technologies, Hong Kong) rotating drum collector rotating at 500 rpm. For the fabrication of the blended fiber mats, electrospinning was performed using two horizontally opposed programmable syringe pumps so that the corresponding spinning solutions were co-electrospun in an antiparallel setup to ensure a homogeneous blending of the polymer fibers. Temperature and relative humidity were set at 21 ± 2 °C and 60 ± 5%, respectively. Electrospinning was performed with the solution feeding rate, applied voltage and tip-to-collector distance fixed appropriately in order to obtain the polymeric micro-/nanofibers.

All spinning solutions were prepared by dissolving the corresponding polymers and EchA at appropriate organic solvent systems at room temperature under stirring for 24 h to ensure their homogeneity. Appropriate quantities of EchA were added to each polymer solution so as to result in a 10% *w/w* (weight to matrix weight) final concentration of EchA. Specifically, the spinning solution of PCL-EchA was prepared by dissolving PCL at a concentration of 12% *w/v* in DCM:DMF 8:2 *v/v* followed by the addition of EchA at a concentration of 1.333% *w/v*. The spinning solution of PVP-EchA was prepared by dissolving PVP at a concentration of 12% *w/v* in ethanol followed by the addition of EchA at a concentration of 1.333% *w/v*. The [PCL-PVP(1:3)]-EchA spinning solution was prepared by dissolving PCL at a concentration of 3% *w/v* and PVP at a concentration of 9% *w/v* in DCM:EtOH 7:3 *v/v*, followed by the addition of EchA at a concentration of 1.333% *w/v*.

For the fabrication of the PCL-EchA and PVP-EchA fiber mats, the PCL-EchA and PVP-EchA spinning solutions were separately electrospun with the solution feeding rate, applied voltage and tip-to-collector distance fixed at 3 mL/h, 25 kV and 15 cm, respectively. For the fabrication of the blended PCL-EchA/PVP-EchA (1:3), PCL-EchA/PVP-EchA (1:1) and PCL-EchA/PVP-EchA (3:1) fiber mats, the PCL-EchA and PVP-EchA spinning solutions were co-electrospun in an antiparallel setup to ensure a homogeneous blending of the PCL and PVP polymer fibers at different ratios. Electrospinning was performed with the applied voltage and tip-to-collector distance fixed at 25 kV and 15 cm, respectively, with the syringes mounted on two horizontally opposed programmable syringe pumps. For the fabrication of the PCL-EchA/PVP-EchA (1:3) fiber mat, the feeding rate of the PCL-EchA spinning solution was adjusted to 1.5 mL/h, whereas the feeding rate of the PVP-EchA spinning solution was fixed at 4.5 mL/h. For the fabrication of the PCL-EchA/PVP-EchA (1:1) fiber mat, the feeding rates of the PCL-EchA and PVP-EchA spinning solutions were both adjusted to 3 mL/h. For the fabrication of the PCL-EchA/PVP-EchA (3:1) fiber mat, the feeding rate of the PCL-EchA spinning solution was adjusted to 4.5 mL/h, whereas the feeding rate of the PVP-EchA spinning solution was fixed at 1.5 mL/h. For the fabrication of the composite [PCL-PVP(1:3)]-EchA fiber mat, the [PCL-PVP(1:3)]-EchA spinning solution was electrospun with the solution feeding rate, applied voltage and tip-to-collector distance fixed at 3 mL/h, 25 kV and 15 cm, respectively.

### 3.5. Scanning Electron Microscopy (SEM)

For the morphological characterization of the micro-/nanofibers, the samples were precoated with a conductive layer of sputtered gold and examined using a PhenomWorld (Thermo Fischer Scientific, Waltham, MA, USA) desktop scanning electron microscope with a tungsten filament (10 kV) and a charge-reduction sample holder. The average fiber diameter was determined using the embedded image analysis software (Phenom Pro Suite/Fibermetric) by evaluating at least 100 measurements per sample.

### 3.6. Fourier Transform Infrared Spectroscopy (FT-IR)

FT-IR spectra were recorded using the attenuated total reflection (ATR) method on a Bruker Alpha II (Billerica, MA, USA) FT-IR spectrometer.

### 3.7. Ultraviolet-Visible Spectroscopy (UV-Vis)

UV-Vis spectra were recorded on an Infinite M200 PRO TECAN plate reader (Männedorf, Zürich, Switzerland). The wavelength range was 230-500 nm, and EchA was detected in the samples via short-lived signals (flash = 2) with steps equal to 2. Calibration curves of EchA were prepared for each buffer medium (pH 1.2, 4.5, 6.8 and 7.4). The calibration curve concentrations ranged from 0.5 to 50 μg/mL of EchA and were prepared using appropriate volumes of a starting stock solution of EchA in methanol diluted with the respective buffer medium.

### 3.8. Thermogravimetric Analysis (TGA)

Thermogravimetric analyses (TGA) were performed using a TGA 55 Thermogravimetric Analyzer (TA Instruments, New Castle, DE, USA) from 40 to 600 °C at a heating rate of 10 °C/min under a 25 mL/min nitrogen flow with the sample weight, sample temperature and heat flow recorded continuously.

### 3.9. Differential Scanning Calorimetry Analysis (DSC)

Differential scanning calorimetry (DSC) analyses were conducted using a Discovery DSC 25 Thermal Analyzer (TA instruments, New Castle, DE, USA). Sealed samples of 6–7 mg in aluminum pans were heated from 40 to 370 °C for the fibrous scaffolds and from 40 to 270 °C for neat EchA at a constant rate of 10 °C/min under a 50 mL/min nitrogen flow.

### 3.10. Dissolution Studies

Dissolution tests for the micro-/nanofibrous patches and neat EchA were performed in three different media using a Vankel 750D dissolution apparatus with the paddle method. The experiments were carried out in a 500 mL final volume of buffer at 37 °C and 50 rpm. The patches were introduced in capsule sinkers, while cellulose capsules were employed for neat EchA to avoid floating of the material during the experiment. Specifically, 20 mg (approx. 4 cm^2^) of patch (containing 2 mg of EchA) or 2 mg of neat EchA were dispersed in 500 mL of HCl 0.1 M (pH 1.2), citric buffer 0.1 M (pH 4.5) or phosphate buffer 0.1 M (pH 6.8). At defined time intervals (5, 10, 15, 20, 30, 45, 60, 120 and 180 min for the dissolution study at pH 1.2 and 5, 10, 15, 20, 30, 45, 60, 120, 180, 240, 300 and 360 min for the dissolution study at pH 4.5 and 6.8), a 3 mL sample was withdrawn from the dissolution medium of each container and replaced with equal volume of fresh dissolution medium. The withdrawn samples were filtered via regenerated cellulose filters (Whatman, Spartan syringe filters, 0.45 μm) to remove undissolved EchA before the absorbance measurements, using 1 mL for each filter’s saturation. The filtered volume was transferred in a UV transparent Corning 96 flat plate, and the absorbance of EchA was measured at 470 nm using an Infinite M200 PRO TECAN (Männedorf, Zürich, Switzerland) plate reader. The dissolution experiments for each patch and neat EchA were run in triplicate at the three tested pH values.

### 3.11. Permeability Studies

The small intestine of young rabbits (*Oryctolagus cuniculus*) was selected as the most appropriate tissue for the ex vivo diffusion experiments. The duodenum of small intestine was extracted on the day of the experiment from rabbits collected from a local slaughterhouse (Athens, Greece). For the isolation of the intestine tissue, a surgical scissor was used to remove the mesentery and cut the intestine on both sides of the duodenum area. Then, a longitudinal section of the isolated part of the intestinal tract allowed for the thorough cleansing of the tissue with water. The serosa and the muscle layers were gently removed using surgical forceps. During the isolation process, the intestinal mucosa was kept hydrated with saline solution. The extracted intestine was cut into squares of a 1 cm^2^ surface to cover completely the Franz cells’ diffusion area (0.636 cm^2^). Each intestine section was mounted between the donor and receptor compartments of the Franz diffusion cell (Crown Glass, Somerville, MA, USA), with the mucosal interior side facing the donor. To assure the proper cell assembly and the integrity of the mucosa, the receptor compartment was filled with phosphate buffer saline (PBS) solution to check that no liquid could pass to the empty donor due to inappropriate mounting or lack of tissue integrity. The Franz cells were assembled by filling the receptor compartment with 5 mL of PBS (pH 7.4) and the membrane was mounted between the receptor and donor compartments. A magnetic stirrer was added into the receptor, and the two parts were kept together with a metal clamp. The assembled system was allowed to equilibrate at 37 °C for 15 min. Then, 2 mg of each patch (containing 0.2 mg of EchA) or 0.2 mg of neat EchA were placed in the donor compartment and were wetted with 1 mL of the pH 6.8 buffer to resemble the intestinal conditions. Both the donor and the receptor compartments were covered with Parafilm^®^ to prevent evaporation. All experiments lasted for 24 h. At specific time intervals (1, 2, 3, 4 and 24 h), 0.5 mL were sampled from the receptor compartment and replaced by an equal volume of fresh PBS. The absorbance of the samples was measured on an Infinite M200 PRO TECAN (Männedorf, Zürich, Switzerland) plate reader. At the end of the experiment, the residual formulation in the donor compartment was quantitatively collected and diluted to determine the remaining EchA and calculate the mass balance. The amount accumulated in the tissue was recovered by comminuting the mucosa with a surgical blade and homogenizing it with a small pestle three times, using 300 μL of water for 30 sec each time. Then, it was further homogenized with 300 μL of methanol for 30 sec. After homogenization, the extract was diluted and centrifuged before the absorbance was measured. The amounts of EchA recovered from the mucosa, receptor and donor compartments allowed for the calculation of the mass balance.

## 4. Conclusions

In the present study, we investigated the development of new pharmaceutical forms of EchA through its incorporation into polymeric micro-/nanofibers. EchA was successfully loaded into electrospun micro-/nanofibrous matrices composed of PCL or/and PVP in various combinations. The fabricated matrices were characterized using SEM, FT-IR, TGA and DSC analyses, and the release profile of EchA from the matrices was evaluated in vitro at three different pH values (1.2, 4.5 and 6.8), simulating the different conditions of the gastrointestinal tract. The dissolution studies revealed variable release profiles of EchA from each polymeric matrix in the different pH environments. Subsequently, ex vivo permeation experiments in the duodenum of the small intestine of young rabitts showed that the incoproration of EchA into the electrospun fibers enhanced its permeation across the duodenum barrier.

According to the obtained results, electrospun polymeric micro-/nanofibers represent a promising carrier for EchA that can control its release either in the stomach or the intestine, while at the same time increasing the solubility and stability of the bioactive compound. The selection of the polymers used as carriers of EchA can affect the release profile, thus allowing for the preparation of micro-/nanofibrous patches tailored according to the desired specifications/properties for different routes of administration and different therapeutic targets, offering the potential for targeted delivery. For example, when EchA should be released in the highly acidic pH environment of the stomach, polymers soluble in acidic pH could be blended with polymers of variable solubility in order to achieve the desired release profiles. In contrast, when EchA should be released in the more alkaline environment of the duodenum and the other parts of the small intestine or in the colon, polymers insoluble in acidic but soluble in neutral to slightly basic media should be selected and blended with polymers of variable solubility in order to achieve prolonged release profiles.

## Data Availability

The data presented in this study are available in the present article.
